# Lysosomes at the Crossroads of Cell Metabolism, Cell Cycle, and Stemness

**DOI:** 10.3390/ijms23042290

**Published:** 2022-02-18

**Authors:** Ada Nowosad, Arnaud Besson

**Affiliations:** 1 Molecular, Cellular and Developmental Biology Department (MCD), Centre de Biologie Intégrative (CBI), University of Toulouse, CNRS, UPS, 31062 Toulouse, France; adanowosad89@gmail.com; 2 Department of Oncology, KULeuven, Laboratory for Molecular Cancer Biology, Center for Cancer Biology, VIB, 3000 Leuven, Belgium

**Keywords:** lysosome, cell metabolism, cell cycle, stemness, cell signaling, nutrient sensing, self-renewal, quiescence, mTOR, CDK

## Abstract

Initially described as lytic bodies due to their degradative and recycling functions, lysosomes play a critical role in metabolic adaptation to nutrient availability. More recently, the contribution of lysosomal proteins to cell signaling has been established, and lysosomes have emerged as signaling hubs that regulate diverse cellular processes, including cell proliferation and cell fate. Deciphering these signaling pathways has revealed an extensive crosstalk between the lysosomal and cell cycle machineries that is only beginning to be understood. Recent studies also indicate that a number of lysosomal proteins are involved in the regulation of embryonic and adult stem cell fate and identity. In this review, we will focus on the role of the lysosome as a signaling platform with an emphasis on its function in integrating nutrient sensing with proliferation and cell cycle progression, as well as in stemness-related features, such as self-renewal and quiescence.

## 1. Introduction

In the early 1950s, Christian de Duve identified intracellular compartments containing acid hydrolases acting on a broad range of carbon-containing macromolecules. He decided to call them lysosomes, meaning digestive body, as a reference to their digestive functions [1]. Although only five hydrolases were initially reported, lysosomes contain approximately 60 different types of enzymes, including proteases, nucleases, glycosidases, and lipases, which break down proteins, nucleic acids, sugars, and lipids, respectively [1,2,3]. The most studied group of lysosomal hydrolases is cathepsins, which convert proteins into amino acids in the lysosomal lumen [4,5]. All lysosomal hydrolases are activated by an acidic pH within the lumen (pH ≈ 4.5–5). Acidification of the lysosomal matrix is mediated by v-ATPase (vacuolar-type H^+^-ATPase), which acts as a proton pump in lysosome membranes, actively transporting H_3_O^+^ ions from the cytosol into the lumen [6]. A similar system is used in all other acidic cellular organelles, such as endosomes, some compartments of the Golgi apparatus, and many transport and secretory vesicles [4]. Furthermore, the luminal side of lysosomal membranes is protected from autodigestion by a continuous layer of highly glycosylated membrane proteins, of which the most abundant group is the LAMPs (lysosome-associated membrane proteins) [7].

Most hydrolases are targeted to lysosomes from the trans-Golgi network (TGN) by a mannose-6-phosphate (M6P)-dependent transport. M6P is conjugated to N-linked oligosaccharides on hydrolases and recognized in the TGN by M6P receptors (MPRs), which contribute to their packaging in clathrin-coated vesicles that bud from the TGN. These vesicles then fuse with early endosomes and eventually mature into late endosomes and lysosomes. Following vesicle acidification, M6P is cleaved from receptors, which are then recycled in retromer-coated vesicles [4,5]. Two MPRs function in the delivery of newly synthesized hydrolases to lysosomes: the ∼46 kDa cation-dependent MPR (CD-MPR) and the ∼300 kDa cation-independent MPR (CI-MPR). CI-MPR also acts as an insulin-like growth factor 2 receptor (IGF2R) and is present at the cell surface, where it mediates the internalization and targeting of IGF2 to lysosomes [8]. Additionally, M6P-independent pathways for lysosomal sorting exist. These require the presence of specialized receptors, such as the lysosomal integral membrane protein 2 (LIMP-2/SCARB2) and Sortilin (SORT1). LIMP-2 mediates the delivery of β-glucocerebrosidase (GCase) to lysosomes, whereas Sortilin is implicated in the Golgi-to-lysosome transport of many proteins, including the sphingolipid activator proteins (SAPs), prosaposin (PSAP), GM2 ganglioside activator protein (GM2A), acid sphingomyelinase (ASM), and cathepsins D and H [9].

Lysosomal enzymes digest extracellular material taken up by endocytosis and intracellular components segregated by autophagy [1]. Based on the mechanism delivering autophagic cargo to lysosomes, autophagy is classified into three categories: macroautophagy, chaperone-mediated autophagy (CMA), and microautophagy [10,11]. Macroautophagy (hereafter referred to as autophagy) is the best-studied form of autophagy and is characterized by the presence of double-membrane vesicles called autophagosomes in which cargo is sequestrated and delivered to lysosomes through autophagosome–lysosome fusion. In microautophagy, a small portion of cytoplasm is directly engulfed by an inward invagination of the lysosomal membrane without the requirement of specialized proteins. CMA consists of the selective degradation of soluble cytoplasmic proteins that are recognized by chaperone proteins (usually HSP70) and directly delivered to lysosomes across the membrane after activation of the lysosomal receptor LAMP2. Therefore, in microautophagy and CMA, the cargo is targeted directly to lysosomes without the formation and transport of additional vesicles [11,12,13]. Following autophagic degradation in autolysosomes, digestion products are transported across lysosomal membranes back to the cytoplasm. The efflux of lysosomal amino acids requires the activity of v-ATPase and amino acid transporters [14,15].

Manipulating lysosomal pathways is emerging as a promising therapeutic strategy. Recent approaches allow targeting abundant proteins for lysosomal degradation using CMA following their fusion with a targeting peptide containing a KFERQ motif, which is present on all CMA substrates [16]. Another strategy is using lysosome-targeting chimeras (LYTACs), which consist of a protein-targeting moiety (small molecule or antibody) conjugated to glycopeptide ligands for CI-MPR, ensuring the transport of glycosylated proteins to lysosomes. While the CMA approach is designed to induce the proteolysis of intracellular proteins, LYTACs allow targeting both extracellular and membrane-associated proteins [17].

## 2. MiT Transcription Factors Control Lysosome Biogenesis

**Figure 1:**
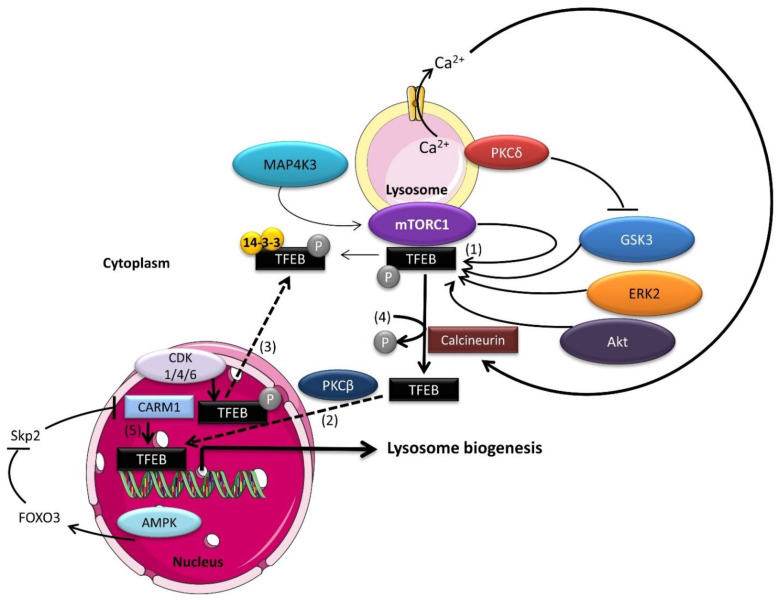
The TFEB regulation network. (1) TFEB phosphorylation by mTORC1, MAP4K3, ERK2, Akt and GSK3 results in TFEB retention in the cytosol, preventing transcription of its target genes. (2) In contrast, PKCβ phosphorylation stabilizes TFEB, increasing its transcriptional activity. (3) In cycling cells, nuclear TFEB is phosphorylated by CDK4/6 in G1 phase and CDK1 in M phase, leading to its nuclear exclusion and inactivation. (4) In stress conditions, the cytoplasmic phosphatases Calcineurin and PP2A dephosphorylate TFEB in response to increased Ca^2+^ or reactive oxygen species (ROS), respectively, leading to TFEB nuclear localization and expression of lysosomal and autophagy-related genes. (5) In glucose-starved cells, nuclear AMPK promotes TFEB-dependent transcription of autophagy and lysosomal genes by stabilizing its coactivator, CARM1.

The expression of genes encoding proteins required for lysosome formation is under the control of transcription factor EB (TFEB), a basic helix–loop–helix leucine zipper protein of the microphthalmia/transcription factor E (MiT/TFE) family, comprising MITF/TFEF, TFEB, TFE3, and TFEC [18,19]. All MiT/TFE members bind to the CLEAR (coordinated lysosomal expression and regulation) DNA motif within the proximal promoters of lysosomal and autophagy genes [18]. TFEF, TFEB, and TFE3 drive expression of their target genes via their acidic activation domain. In contrast, TFEC lacks this acidic domain and binds to other MiT family members, inhibiting their function [20]. While MITF/TFEF, TFEB, and TFE3 have all been shown to regulate the autophagy-lysosomal pathway [19,21,22,23], TFEB overexpression is sufficient to induce lysosome synthesis and autophagy flux [24]. TFEB activity is regulated by phosphorylations that control its subcellular localization and affinity for binding partners. TFEB contains a nuclear export signal (NES), whose phosphorylation on nearby serines (S122, S134, S138, and S142) is a major determinant of cytoplasmic-nuclear shuttling [25] (Figure 1). In addition, TFEB phosphorylation on S211 by mTORC1 promotes binding to 14-3-3 and cytoplasmic retention [26,27]. Several kinases targeting TFEB have been identified. While ERK2 [28], GSK3β [29], mTORC1 [26,27], Akt [30], MAP4K [31], CDK4/6 [21], and CDK1 [32] mediated phosphorylation inhibits TFEB transcriptional activity by preventing its translocation to the nucleus [21,27,28,29,30,31], its phosphorylation by PKCβ in osteoclasts stabilizes TFEB and promotes transcription of its target genes [33]. Conversely, stress-induced protein phosphatases promote TFEB dephosphorylation and activation. For instance, nutrient withdrawal stimulates the release of the lysosomal Ca^2+^ pool through the cation channel MCOLN1 (mucolipin 1), activating calcineurin and leading to TFEB dephosphorylation and translocation to the nucleus [34] (Figure 1). Similarly, TFEB is activated following oxidative stress by PP2A-mediated dephosphorylation [35]. Recent studies identified CARM1 (co-activator-associated arginine methyltransferase 1) as a TFEB coactivator. Upon glucose deprivation, AMPK (5′AMP-activated protein kinase) activation leads to transcriptional repression of the Skp2 E3 ubiquitin ligase, which is responsible for CARM1 degradation in non-starved cells. In turn, stabilized CARM1 promotes TFEB-driven gene expression, resulting in autophagy induction [36]. Additionally, glucose deprivation leads to mTORC2 activation and subsequent phosphorylation of Akt, which, in turn, inactivates GSK3β, leading to nuclear accumulation of TFEB and expression of autophagy-related genes [25]. Thus, when nutrients are plentiful, TFEB relocalizes to the cytoplasm within minutes [37,38], whereas ZKSCAN3 (zinc finger with KRAB and SCAN domains 3) enters the nucleus to act as a transcriptional repressor on gene promoters of the autophagy–lysosome network [39]. Thus, ZKSCAN3 acts in conjunction with TFEB to adapt the response of cells to their metabolic environment [39]. As the dynamics of the signaling networks controlling the content and activity of the lysosomal compartment are unveiled, the crucial role of lysosomes in metabolic plasticity, allowing cells to adapt to environmental changes dictated by intracellular and extracellular cues, becomes evident.

## 3. The Lysosome as a Signaling Hub

An increasing number of studies show that lysosomal membranes play a central role in coordinating the cellular response to nutrients [40]. The discovery that lysosomes act as a signaling platform for the nutrient-sensing machinery and that mTORC1, a master regulator of cell metabolism, is activated on the lysosomal surface in response to amino acids and glucose has generated great interest.

**Figure 2:**
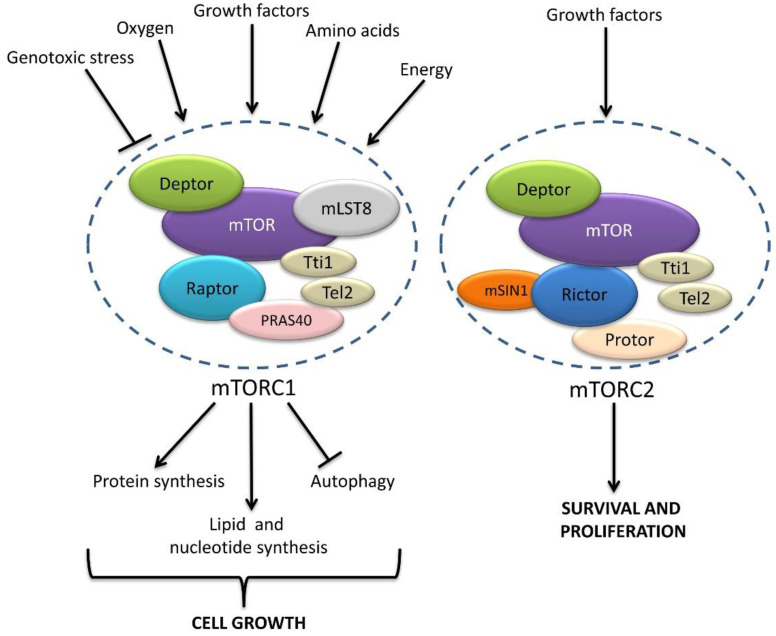
Schematic representation of mTORC1 and mTORC2. mTORC1 and mTORC2 share the catalytic mTOR kinase and the regulatory subunits Deptor, mLST8, and Tti/Tel2. In addition, mTORC1 consists of Raptor and PRAS40, conferring mTORC1 the ability to respond to a broad range of environmental signals, including DNA damage and growth factors, as well as oxygen, nutrient, and energy levels. mTORC1 phosphorylates multiple targets to control the balance between anabolic (leading to cell growth) and catabolic (leading to energy production) processes. The mTORC2 complex contains the subunits Rictor, Sin1, and Protor and mediates the response to growth factors, contributing to cell proliferation and survival.

Further, mTOR (mammalian target of rapamycin) is a 289 kDa serine/threonine kinase that belongs to the PI3K-related kinase superfamily, and mTOR nucleates two distinct complexes, named mTOR complex 1 (mTORC1) and mTOR complex 2 (mTORC2), having different binding partners and substrate specificity (Figure 2) [41,42,43]. It contains three core components: mTOR, Raptor (regulatory protein associated with mTOR), and mLST8 (mammalian lethal with Sec13 protein 8, also known as GßL), and two inhibitory subunits, PRAS40 (proline-rich Akt substrate of 40 kDa) and DEPTOR (DEP domain containing mTOR interacting protein) [41], as well as the Tti1/Tel2 complex, which is important for the assembly and stability of the mTORC1 complex [43]. The mTORC1 activity is inhibited by rapamycin in a FKBP12-dependent manner [44,45]. Earlier studies showed that rapamycin acts as an allosteric inhibitor of the mTOR kinase domain [46,47,48], but more recent findings revealed that binding of rapamycin-FKBP12 to mTORC1 leads to mTORC1 disassembly, thereby preventing the phosphorylation of some, but not all, of its downstream targets [49]. The main role of mTORC1 is to adapt the cellular metabolism to the environment. Thus, mTORC1 responds to a broad spectrum of cues, such as growth factors, energy, oxygen, DNA damage, and amino acids, to regulate cell growth [41,50]. The coordination of all these inputs requires a complex signaling network in which mTORC1 acts as a hub that orchestrates the cellular response to environmental changes.

Additionally, mTORC2 contains mLST8 and DEPTOR but relies on different regulatory subunits, Rictor (rapamycin insensitive companion of mTOR), mSin1, and Protor1/2 [41], and mTORC2 is mainly activated by growth factors and promotes cell survival and proliferation via the regulation of the cytoskeleton and PI3K signaling [41,44,45]. The mTORC2 and Akt activity also participate in the regulation of lysosome positioning [51]. Although mTORC2 was previously thought to be resistant to rapamycin, recent studies revealed that prolonged exposure to rapamycin results in inhibition of mTORC2 kinase activity [52,53,54].

## 4. The Lysosome as a Platform for the Nutrient-Sensing Machinery 

The GTPase Rheb (Ras-homolog enriched in brain) is a critical activator of mTORC1 [55,56,57,58]. Rheb is anchored on lysosomes by the presence of a lipid moiety, a farnesyl, on its C-terminus. Thus, mTORC1 requires translocation to lysosomes to be activated. After amino acid stimulation, cytoplasmic mTORC1 is recruited to lysosomes within minutes by Rag GTPases [59]. The identification of Rags as a major determinant of mTORC1 localization was a crucial step in understanding the mechanism by which mTORC1 responds to amino acids [59,60]. Unlike most GTPases that function as monomers, Rags form heterodimers between RagA or -B and RagC or -D. Shortly after amino acid stimulation, the Rag complex is activated, which corresponds to RagA/B being GTP-loaded, while RagC/D is GDP loaded [59,60,61]. The binding of GTP to one of the subunits causes a conformational change that prevents GTP loading or triggers GTP hydrolysis in the second subunit [62]. In their active conformation, Rags recruit mTORC1 to the lysosomal surface by binding to the mTORC1 regulatory subunit Raptor [59,63,64]. Importantly, Rags also play a critical role in localizing MiT transcription factors to lysosomal membranes in nutrient rich conditions, thus bringing them in proximity of mTOR for phosphorylation and inhibition. Indeed, while Raptor binds extensively to RagA/B-GTP, TFEB and MITF interact with RagC/D-GDP [64,65]. Thus, Rag GTPases appear to play an essential role in the response of cells to amino acid stimulation after starvation. Nevertheless, recent studies found that, in nutrient rich steady state conditions, RagC/D binding to TFE3 or TFEB regulate embryonic stem cell differentiation and myelination, respectively [66,67].

Since Rags lack lipid modifications to anchor them to lysosomal membranes, they rely on other proteins to act as a scaffold for the Rag/mTORC1 complex [63]. This scaffold, named Ragulator, is a multiprotein complex comprising five subunits, p18/LAMTOR1, p14/LAMTOR2, MP1/LAMTOR3, C7orf59/LAMTOR4, and HBXIP/LAMTOR5 [63,68]. LAMTOR1 acts as a scaffold for the other Ragulator subunits, and it is myristoylated and palmitoylated, thus anchoring the complex on lysosomal membranes [63]. On lysosomes, Ragulator is assembled sequentially from LAMTOR1, -5, and -4 trimers [69]. The crystal structure of human Ragulator revealed that LAMTOR2 and -3 and LAMTOR4 and -5 form dimeric sub-complexes that are surrounded by LAMTOR1, which displays a belt-like shape, wrapping around the other four subunits [70].

Although Rags were initially described as being constitutively located on the lysosomal surface [63], further studies have shown that Rags cycle between cytoplasm and lysosome membranes and that their shuttling is regulated by amino acids availability, which promotes their dissociation from Ragulator [71]. Upon amino acid stimulation, Rags are rapidly released from lysosomal membranes to reduce the time that mTORC1 spends in direct contact with its activator, Rheb, preventing mTORC1 hyperactivation [71].

Rheb activity is negatively regulated by the tuberous sclerosis complex (TSC), which acts as a GTPase activating protein (GAP) towards Rheb [72,73,74]. TSC is a heterotrimeric complex containing TSC1 (tuberous sclerosis 1 protein/hamartin), TSC2 (tuberous sclerosis 2 protein/tuberin), and TBC1D7 (TBC1 domain family member 7). The C-terminal domain of TSC2 stimulates GTP hydrolysis by Rheb, while the other two subunits stabilize TSC2 and enhance its GAP activity [55,75,76,77]. The TSC complex is regulated by growth factors, with tyrosine kinase receptor activation leading to PI3K activation and increased PIP_3_ (phosphatidylinositol (3,4,5)-triphosphate) levels. The serine/threonine kinases PDK1 (PDPK1, 3′-phosphoinositide-dependent kinase 1), and Akt bind to PIP_3_ on the plasma membrane, promoting Akt activation by PDK1. In turn, Akt phosphorylates TSC2, turning off its GAP activity towards Rheb [41,42,50,78,79,80]. Thus, growth factor signaling activates mTORC1 via TSC inhibition. Conversely, AMPK phosphorylates TSC2, increasing its activity and thereby reducing mTORC1 signaling under low energy conditions [81]. Although the regulation of the TSC complex by growth factors and carbohydrates signaling is well established, its regulation by amino acid levels is less clear. Recent studies showed that TCS2 is recruited to lysosomal membranes in response to either amino acid or growth factor withdrawal, promoting the interaction between TSC and Rheb [82,83,84]. The translocation of TSC to lysosomes, where it is anchored through an interaction with G3BP1/2 (Ras GTPase-activating protein-binding proteins 1 and 2) [85], is mediated by Rag GTPases and Ragulator [63,82]. Therefore, the Rag/Ragulator complex acts as a scaffold, coordinating both positive and negative inputs on mTORC1 signaling, promoting mTORC1 activation when nutrients are abundant and by recruiting TSC2 that suppresses Rheb activity towards mTORC1 during starvation [63,82,83].

Rheb activity is also controlled by post-translational modifications. In response to energy starvation, Rheb is phosphorylated by p38 regulated/activated kinase (PRAK) at S130, independent of AMPK, preventing Rheb-mediated mTORC1 activation [86]. Other studies highlight the role of ubiquitination in the regulation of mTORC1. For instance, in starved cells, the lysosomal E3 ligase RNF152 targets RagA for K63-linked ubiquitination, generating an anchor on RagA to recruit its inhibitor, GATOR1, resulting in mTORC1 inactivation [87]. The presence of amino acids reduces the interaction between RNF152 and RagA, allowing mTORC1 activation on lysosomes [87]. Conversely, growth factors induce USP4 (ubiquitin specific peptidase 4)-mediated deubiquitination of Rheb and mTORC1 activation [88]. In contrast to growth factor-induced mTORC1 stimulation, ubiquitination of TSC2 at K8 does not contribute to mTORC1 regulation by amino acids [89]. Instead, Rheb is polyubiquitinated following amino acid stimulation, which promotes Rheb binding to mTORC1 and its activation. When amino acid levels are low, Rheb is maintained in a hypo-ubiquitinated state by the deubiquitinase Ataxin 3 (ATXN3), which is recruited to lysosomes by inactive Rag heterodimers [89]. On the other hand, amino acid-mediated Rag activation releases ATXN3 from lysosomes, promoting Rheb polyubiquitination and positively regulating mTORC1 activity [89]. Further studies to identify the ubiquitination sites and the nature of ubiquitin chain linkage and length may help to clarify the role of Rheb ubiquitination in mTORC1 signaling. The enrichment of Rheb on lysosomal membranes provides a rationale behind the translocation of mTORC1 to lysosomes prior to its activation [63]. However, several studies challenge this model with observations that Rheb may be present on non-lysosomal endomembranes, such as Golgi [90] or ER [91], and showing that transient interactions between Rheb-positive organelles and lysosomes to which mTORC1 is recruited via Ragulator/Rag are sufficient to activate mTORC1 [90,91,92].

**Figure 3:**
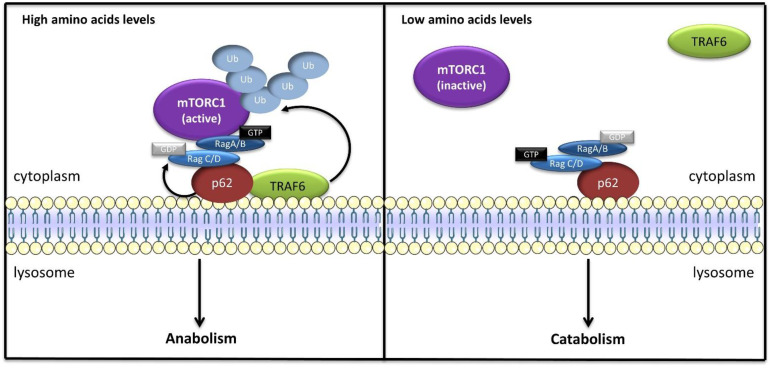
p62-dependent mTORC1 activation. Under amino acid-rich conditions, lysosomal p62 binds to Rags and promotes the assembly of active Rag heterodimers, which, in turn, recruit mTORC1 to lysosomes and promote its activation. Additionally, p62 recruits TRAF6, which promotes mTORC1 polyubiquitination, leading to induction of mTORC1 signaling.

Although Ragulator appears to be required for Rag anchoring on lysosomes, p62/SQSTM1 may act as an alternative lysosomal scaffold for Rags in response to amino acids [93]. Further, p62 is a multifunctional adaptor protein that interacts with many different signaling proteins, notably acting as a signaling hub in the atypical PKC pathway, to regulate multiple cellular processes, such as cell survival, inflammation, apoptosis, and autophagy [93,94], and p62 binds to Rag GTPases and promotes the assembly of active RagB-GTP/RagC-GDP heterodimers, but, surprisingly, p62 does not appear to interact with any subunit of Ragulator, suggesting that p62/Rags and Ragulator/Rags exist as distinct complexes [93]. Interestingly, p62 was shown to interact with Raptor, promoting mTORC1 recruitment to lysosomal membranes and its activation in response to amino acids (Figure 3) [93]. Additionally, p62 recruits TRAF6 (TNF receptor associated factor 6), which induces K63-linked polyubiquitination of mTOR on the lysosomal surface in amino acid-stimulated cells, promoting mTORC1 activity [95,96].

Further insight into how cells sense amino acid levels to regulate mTORC1 came with the discovery that v-ATPase acts as an intracellular amino acids sensor and as a component of the Ragulator–Rag complex on lysosomal membranes [97,98]. According to the current inside-out model, extracellular amino acids are transported via a combination of endocytosis and uptake to accumulate within the lysosomal lumen [41,97,99,100,101]. In response to increased luminal amino acid levels, the interaction between v-ATPase and the Ragulator–Rag complex is weakened. In turn, Ragulator allosterically promotes GTP release from RagC/D [97]. Concomitantly, the amino acid transporter SLC38A9 (solute carrier family 38 member 9) acts as a GEF towards RagA/B, promoting exchange of GDP for GTP, completing the activation of the Rag heterodimer and recruiting mTORC1 to lysosomes [68,98,102]. Conversely, amino acid deprivation strengthens the v-ATPase/Ragulator interaction and prevents GTP loading on RagA/B [68]. An additional level of regulation involves the amino acid-dependent assembly of v-ATPase [103]; v-ATPase is composed of two multi subunit domains, the peripheral V_1_ domain that catalyzes ATP hydrolysis and the membrane-associated V_0_ domain responsible for proton translocation [104], whose assembly is required for vesicle acidification [105,106]. Assembly of the V_0_/V_1_ domains increases during amino acid starvation, promoting v-ATPase activity and lysosome acidification [103]. Interestingly, glucose deprivation has the opposite effect on v-ATPase assembly, attenuating its proton pump activity, suggesting distinct regulations of the adaptive response to amino acid or sugar withdrawal [107,108].

Amino acid concentration within lysosomes is regulated by amino acid transporters that control the efflux and influx of amino acids between the lysosomes and the cytoplasm. For instance, SLC36A1/PAT1 (solute carrier family 36 member 1), which localizes to lysosomes and interacts with RagC, controls the export of small neutral amino acids (Gly, Ala, and Pro) to the cytoplasm [99,100,109]. Other transporters include SLC7A5/LAT-1 and SLC1A5, whose lysosomal localization is driven by DRAM-1 proteins [110]. Finally, SLC38A9, which is part of the lysosomal v-ATPase/Ragulator/Rag super-complex, is involved in sensing arginine, glutamine, and lysine [15,98,100,101,111]. The binding of Arg to SLC38A9 in the lysosomal lumen is required for its ability to sense other amino acids [15], as well as to promote its GEF activity towards RagA/B [102]. Additionally, some transporters are directly involved in modulating mTORC1 signaling [112,113]. This particular group of amino acid transporters is called transceptors, and their role as amino acid sensors is reviewed elsewhere [112,113,114]. Interestingly, recent findings revealed that SLC38A9 also couples cholesterol metabolism to mTORC1. SLC38A9 senses increased cholesterol concentrations in the lumen independent of arginine and promote Rag-dependent mTORC1 recruitment and activation. In contrast, NPC1 (Niemann-Pick C1 protein), which controls cholesterol export from lysosomes, interacts with SLC38A9 and inhibits mTORC1 via its sterol transport activity [115].

**Figure 4:**
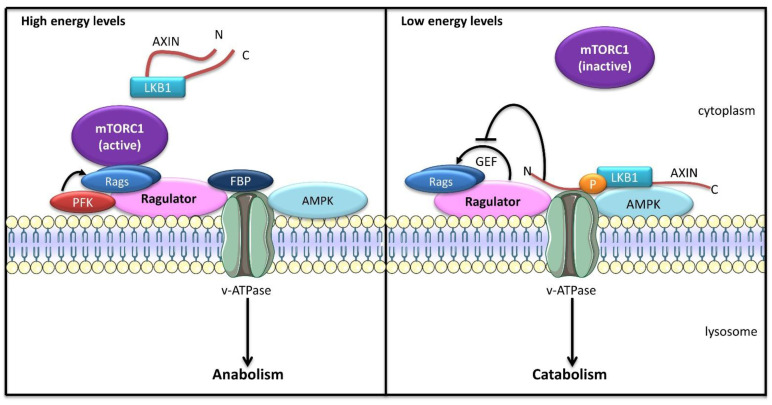
Glucose sensing on lysosomal membrane. In nutrient-rich conditions (high energy), high concentrations of fructose-1,6-bisphosphate (FBP) prevent the translocation of the AXIN/LKB1 complex to Ragulator and the activation of AMPK. Furthermore, the glycolytic enzymes phosphofructokinase 1/2 (PFK) interact with RagB, promoting mTORC1 activation on lysosomal membrane and turning on cell anabolism. In starved cells (low energy), with low FBP levels, lysosomal Ragulator acts as a docking site for the AXIN/LKB1 complex, allowing LKB1-mediated phosphorylation of AMPK and its activation. The recruitment of AXIN to Ragulator inhibits the latter’s GEF activity towards Rags, releasing mTORC1 from lysosomal membrane, thus suppressing its pro-anabolic activity.

The *inside-out* model of mTORC1 activation was further completed with the discovery that v-ATPase acts as a universal nutrient sensor, responding to both intracellular amino acid and glucose levels [116]. AMPK is a serine/threonine kinase activated in response to low energy status that promotes catabolic pathways, such as autophagy. AMPK may localize on lysosomal membranes in vicinity of the Rag/Ragulator/v-ATPase complex. Upon glucose starvation, AXIN and LKB1 are recruited to lysosomes in a Ragulator-dependent manner (Figure 4). LKB1 phosphorylates and activates AMPK, triggering AMPK-mediated metabolic changes. Furthermore, AXIN binding to LAMTOR1 abolishes the GEF activity of Ragulator, preventing Rag activation and mTORC1 recruitment [116]. Translocation of the AXIN/LKB1 complex to lysosomes is induced by metformin, an anti-diabetic drug known to activate AMPK-driven metabolism [117]. In contrast to its canonical mechanism of activation, the lysosomal pool of AMPK is activated independent of the AMP/ATP and ADP/ATP ratios. Instead, low levels of fructose-1,6-bisphosphate (FBP), an intermediate in the glycolysis pathway that is cleaved into dihydroxyacetone phosphate (DHAP) and glyceraldehyde-3-phosphate by aldolases, mediate AMPK activation. In absence of FBP, unoccupied aldolases promote the formation of the AXIN/LKB1/Ragulator/v-ATPase complex and AMPK activation [118]. Conversely, the presence of FBP leads to the release of AXIN/LKB1 from Ragulator, preventing AMPK activation on lysosomes [118]. Interestingly, DHAP was shown to directly activate mTORC1 on lysosomes in a Rag-dependent manner but independent of AMPK [119]. Furthermore, the glycolytic enzymes PFKFB3 (6-phosphofructo-2-kinase/fructose-2,6-biphosphatase 3) and PFK1 (phosphofructokinase-1) interact with RagB to promote mTORC1 translocation to lysosomes and its activation [120], showing that glycolytic enzymes and intermediates play a direct role in the regulation of cell metabolism.

**Figure 5:**
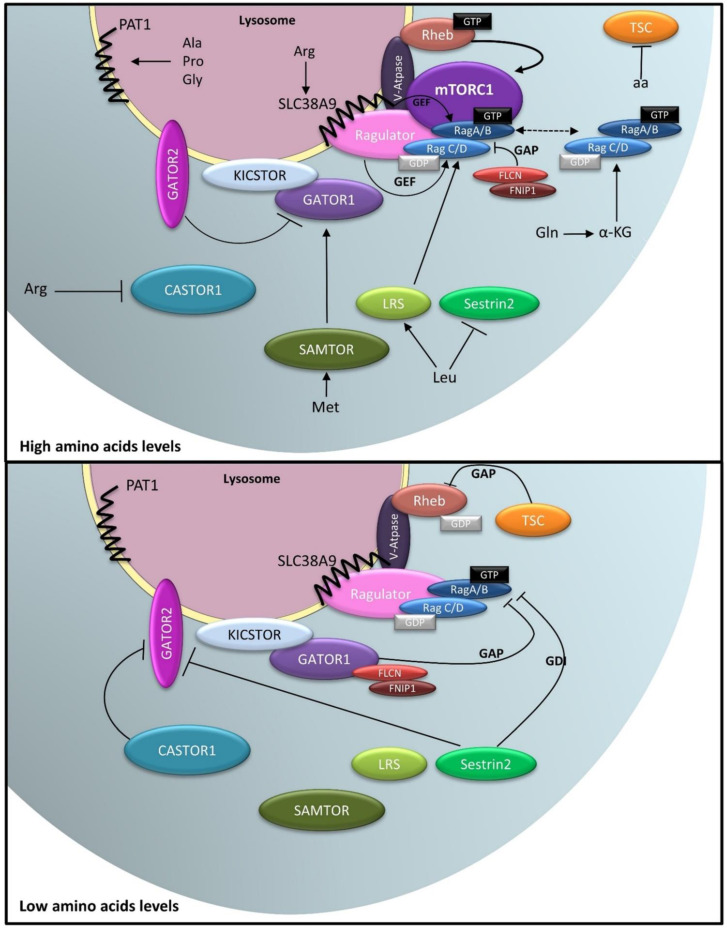
Role of lysosomes in amino acid sensing. Amino acids transporters, such as PAT1 and SLC38A9, regulate amino acids efflux from lysosomes and their concentration in the lysosomal lumen. Lysosomal vATPase acts as an amino acid sensor. Increased amino acid levels weaken the interaction between vATPase and Ragulator, promoting the exchange of GTP for GDP on RagC/D by Ragulator. Additionally, SLC38A9 acts as GEF towards RagA/B, whereas FLCN-FNIP2 exhibits GAP activity towards RagC/D. RagA/B is negatively regulated by GATOR1, whose activity is, in turn, antagonized by GATOR2. In presence of cytoplasmic amino acids, Sestrin2 and CASTOR1 suppress Rags and GATOR2 activities, respectively. KICKSTOR docks GATOR1 on lysosomal surface in an amino acid-independent manner. Met metabolism is sensed by SAMTOR, disrupting the SAMTOR–GATOR1–KICKSTOR complex and preventing mTORC1 inactivation by GATOR1. Furthermore, Rags activity may be controlled directly by Leu and Gln metabolism. Once activated, the Rag complex (RagA/B-GTP/RagC/D-GDP) recruits mTORC1 to lysosomal membranes in close proximity to its upstream activator, Rheb, leading to mTORC1 activation. Within minutes following mTORC1 activation, Rags are released from the lysosomal surface to the cytoplasm, thereby controlling the extent of mTORC1 response.

Cytosolic pathways of amino acid sensing also participate in the lysosomal inside-out model to modulate mTORC1 activity (Figure 5). The GATOR1 and -2 (GTPase activating proteins toward Rags complex 1/2) complexes respond to cytosolic leucine and arginine concentrations to regulate Rag activity [121]. GATOR1 consists of three subunits, DEPDC5 (DEP domain-containing 5) and NPRL2 and -3 (nitrogen permease related-like 2 and-3), in which the DEPDC5 subunit interacts with RagA/B and NPRL2 acts as a GAP for RagA/B, antagonizing Ragulator activity [121,122]. GATOR1 is tethered to lysosomal membranes by the lysosomal complex KICSTOR that consists of four proteins: KPTN, ITFG2, C12orf66, and SZT2 [121,122,123,124]. Although GATOR 1 is present on lysosomes regardless of amino acid levels, its interaction with RagA/B is strengthened by amino acid deprivation, leading to mTORC1 inhibition [121]. In contrast, in presence of leucine, GATOR2, a pentameric complex of WDR59, WDR24, MIOS, SEH1L, and SEC13, which also resides on lysosomes in an amino acid-insensitive manner, suppresses the GAP activity of GATOR1, positively regulating mTOR [101,121,123,125,126,127]. Sestrins (1/2/3) were shown to act as direct leucine sensors in the cytoplasm, connecting cytosolic leucine to the lysosomal mTORC1 machinery [128,129]. In absence of leucine, Sestrins bind to and inhibit GATOR2, allowing GATOR1 to exhibit its GAP activity towards RagA/B [125,126]. Additionally, Sestrins act as GDIs (guanine nucleotide dissociation inhibitors) for Rags, preventing mTORC1 recruitment to lysosomes and its subsequent activation [130]. Finally, Sestrin2 is an ATF4 (activating transcriptional factor 4) target that is induced during prolonged amino acid deprivation to sustain mTORC1 inhibition [131]. Overall, Sestrins negatively regulate mTORC1 via distinct mechanisms when leucine is absent, and, conversely, the presence of leucine disrupts the Sestrin-GATOR2 interaction, leading to mTORC1 activation [128]. GATOR2 is also inhibited by CASTOR1 (cellular arginine sensors for mTORC1) that acts as a sensor for cytoplasmic arginine [132]. CASTOR1 binds to GATOR2 in absence of arginine and dissociates from it upon binding to arginine, thus allowing Rag activation and mTORC1 recruitment to lysosomes [132]. SAMTOR (S-adenosylmethionine sensor upstream of mTORC1) acts as a methionine sensor by binding to the methionine metabolism product *S*-adenosyl-L-methionine (SAM) [133]. In absence of SAM, SAMTOR negatively regulates mTORC1 by interacting with GATOR1 and KICKSTOR. Inversely, binding of SAM to SAMTOR disrupts the SAMTOR–GATOR1–KICKSTOR complex, preventing mTOR inactivation by GATOR1 [133]. Together, the GATOR1 and -2 complexes control the response of mTORC1 to cytoplasmic amino acids by acting upstream of Rag GTPases.

Rag activity is also controlled by the cytoplasmic protein FLCN (folliculin) (Figure 5). In amino acid-deprived cells, FLCN associates with FNIP1 or -2 (folliculin interacting protein 1/2) and is recruited to lysosomes in a GATOR1-dependent fashion [134,135]. The FLCN–FNIP2 complex acts as a GAP towards RagC/D [134,135,136,137]. Lysosomal FLCN-FNIP2 interacts with RagA^GDP^ and, together with Ragulator, blocks the exchange of GDP for GTP on RagA, reducing its GAP activity towards RagC. Upon amino acid stimulation, disassembly of the lysosomal FLCN–FNIP2 complex switches on the GAP activity of FLCN towards RagC, leading to mTORC1 activation [135,137].

Another pathway regulating mTOR upon leucine stimulation is mediated by LARS (leucyl-tRNA synthetase), the enzyme catalyzing the ligation of leucine to its cognate tRNA, which acts as a GAP for RagD, promoting mTORC1 activation [138]. In this way, LARS antagonizes Sestrin2-mediated inhibition of Rag activity [139]. Interestingly, LARS was also reported to activate mTOR in response to leucine independent of Rags via a Vps34-Phospholipase D1 (PLD1)-mTOR pathway [140]. Upon leucine stimulation, LARS activates Vps34, resulting in generation of PI_3_P, which activates PLD1 on lysosomes, catalyzing the hydrolysis of phosphatidylcholine to phosphatidic acid. The latter directly binds to mTOR to modulate its activity [140,141]. Similarly, exogenously supplied fatty acids were shown to activate mTOR via phosphatidic acid [142]. The role of Vps34 in mTORC1 activation is supported by several studies [143,144,145]. According to Hong et al. [145], Vps34-stimulated PI_3_P production on lysosomes leads to the recruitment of FYCO1 to lysosomes and promoting contacts between lysosomal FYCO1 and another PI_3_P effector, protrudin, located on the ER. In turn, this enhances anterograde movement of lysosomes, which is associated with mTORC1 activation. It is not clear how Vps34-mediated mTORC1 activation could be reconciled with the Sestrin2/GATOR2/Rags axis, but it is possible that these two pathways act simultaneously to sense cytoplasmic leucine.

In addition to amino acids, the lysosomal machinery can sense various products and intermediates of amino acid metabolism. For instance, leucine and glutamine are directly sensed by glutaminolysis enzymes. Leucine binds to and activates glutamate dehydrogenase, which mediates glutamine catabolism, resulting in α-ketoglutarate (α-KG) production. α-KG acts as a surrogate for glutamine sensing and stimulates GTP loading on RagB, leading to lysosomal recruitment of mTORC1 and its activation [146,147]. However, glutamine may also be sensed in a Rag-independent manner via a mechanism involving phospholipase D1 [146,147]. Importantly, the source of amino acids appears to determine the pathway triggering mTORC1 activation. While most exogenously acquired amino acids require Rags, amino acids derived through protein degradation in lysosomes activate mTORC1 independent of Rags [148]. Instead, the HOPS (homotypic fusion and vacuole protein sorting) complex, which mediates the fusion of cargo-containing vesicles with lysosomes, is required for mTORC1 activation following the degradation of protein acquired through Ras-driven micropinocytosis [148].

In absence of amino acids, TSC translocates to lysosomes to inhibit Rheb activity, keeping mTORC1 inactive. Arg deficiency is sensed by CASTOR1, which, in turn, binds to GATOR2, releasing GATOR1 from the inhibitory effect of GATOR2. GATOR1 binds to RagA/B and turns on its GAP activity, leading to inactivation of the Rag complex. Additionally, Leu deprivation switches on the GDI activity of Sestrin2 towards RagA/B, preventing mTORC1 recruitment to lysosomal membrane. 

Ala–alanine, Arg–arginine, Gly–glycine, Gln–glutamine, Leu–leucine, Met–methionine, Pro–proline, α-KG–α-ketoglutarate, GEF–guanine nucleotide exchange factor, GAP–GTPase activating protein, GDI–guanine nucleotide dissociation inhibitor. Adapted from [79,83,100].

## 5. Lysosomes and Cell Cycle Regulation 

Proliferating cells must integrate cell growth and cell division signals in order to coordinate metabolic pathways, leading to either mass (anabolic processes) or energy (catabolic processes) production, which are required for cell cycle progression. Therefore, it is not surprising that lysosomes, which act as signaling hubs for major signaling pathways controlling metabolism (mTORC1 and autophagy), play a direct role in cell cycle regulation.

### 5.1. Control of the Cell Cycle by the Lysosomal Machinery

**Figure 6:**
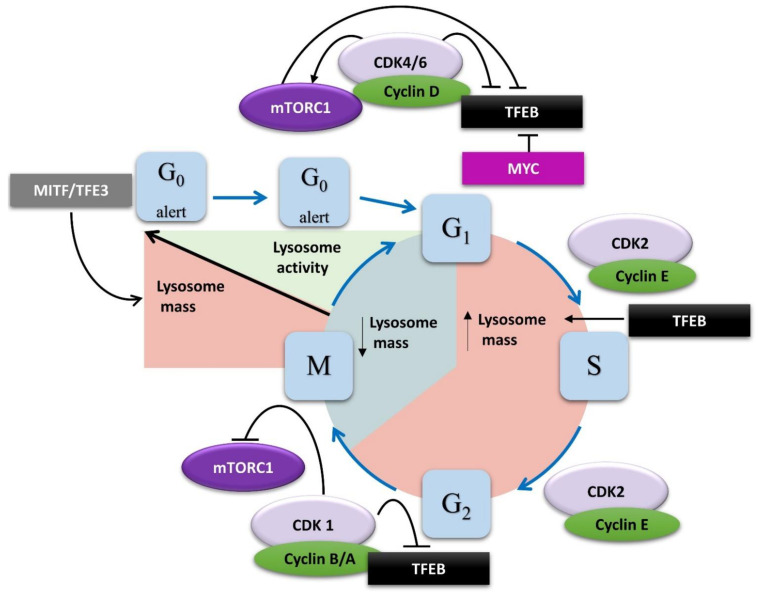
Changes in lysosome mass and activity throughout the cell cycle. Lysosomal gene expression increases in deep quiescence as a result of elevated MITF and TFE3 activity. Priming from deep to shallow quiescence requires activation of lysosomal functions to promote catabolic pathways, providing energy required for cycling. As cells progress through G1 phase, CDK4/6 and MYC inactivate TFEB directly or via mTORC1 activation, decreasing lysosomal content. When cyclin D levels decrease in S phase, TFEB is released from the inhibitory effect of CDK4 and drives lysosomal gene expression, expanding the lysosomal compartment. During M phase, CDK1 inhibits TFEB and mTORC1, decoupling nutrients sensing from cell metabolism to protect genome integrity. As cells progress to anaphase, CDK1 is inactivated, and TFEB resumes lysosome biosynthesis, preparing cells for cytokinesis and mitotic exit.

Cell cycle progression is controlled by sequential activation of cyclin-dependent kinases (CDKs), which promote the transition through the different cell cycle phases (Figure 6). Cells can also exit the cell cycle and enter quiescence, a reversible state characterized by low metabolic activity. Importantly, quiescence is a heterogeneous state as arrested cells can display different molecular signatures and degrees of responsiveness to proliferative signals [149,150]. Recent studies highlight the involvement of the lysosomal pathway in controlling the depth of quiescence in serum-starved fibroblasts [150]. In these cells, RNA-Seq experiments revealed that MITF and TFE3 (but not TFEB) expression increases as quiescence deepens, enhancing lysosome biogenesis. These newly synthesized lysosomes exhibit reduced activity, and their activation is required for transitioning from a deep to a shallow quiescence state and for cell cycle re-entry [150]. Lysosome activation ensures the clearance of mitochondria, which is essential to operate the metabolic switch from oxidative phosphorylation to glycolysis that is observed in many cell types when exiting quiescence [151,152,153]. Similarly, during mitotic arrest, cells use lysosomal pathways to degrade mitochondria, resulting in reduced ATP levels and activation of AMPK [154]. Once activated, AMPK phosphorylates PFKFB3, leading to repression of oxidative phosphorylation and increasing glycolysis, which is required for cell survival during prolonged mitotic arrest [154]. Deregulation of mitochondrial function was also shown to impair the lysosomal compartment, resulting in accumulation of large lysosomes and increased reactive oxygen species (ROS) production [155]. The same phenotype characterizes cells in a deep quiescent state [150,156], suggesting that a mitochondria–lysosome crosstalk coordinates metabolic plasticity and is required for cell cycle re-entry. The lysosomal calcium channel MCOLN1 acts as a ROS sensor on lysosomal membranes, and high ROS levels trigger Ca^2+^ release from lysosomes, promoting TFEB activation and expression of autophagy and lysosomal genes, allowing clearance of mitochondria [157]. Therefore, the rise in intracellular ROS to a critical level may lead to lysosome activation and autophagy induction, and, in turn, the generation of autophagy-derived amino acids activates mTORC1 to prime cells for cell cycle re-entry [158]. Furthermore, recent studies showed that mTORC1 senses mitochondria dysfunctions via AMPK and EIF2AK1 (eukaryotic translation initiation factor 2 alpha kinase 1), which leads to activation of ATF4, driving autophagy-mediated clearance of damaged organelles [159].

In response to DNA damage, p53 induces cell cycle arrest in the G1 phase in an mTORC1- and TFE3/TFEB-dependent manner [160]. Indeed, genotoxic stress inactivates mTORC1, leading to TFEB/TFE3 dephosphorylation and nuclear translocation, where they contribute to p53 stabilization by suppressing Mdm2-dependent p53 degradation [160]. Importantly, RNA-Seq analyses of TFEB/TFE3 double knockout cells exposed to genotoxic stress revealed that expression of key genes controlling the cell cycle is impaired in these cells, including cyclins B1, -B2, and -A2, Aurora B, Survivin, PLK1, and TTK, as well as genes implicated in chromosome segregation and cytokinesis [160]. This confirms that TFEB and TFE3 are directly involved in regulating cell cycle checkpoints and cell cycle progression in response to stress.

### 5.2. Regulation of Lysosomes by the Cell Cycle Machinery 

As cells enter the G_1_ phase, CDK4/6 complexes phosphorylate TFEB, leading to its nuclear export and inactivation (Figure 1 and Figure 6) [21], and CDK4/6 inhibition increases lysosome biogenesis in breast cancer cells [161,162]. This mechanism may explain the induction of deep quiescence/senescence observed in breast cancer [162] and neuroblastoma cells [163] treated with CDK4/6 inhibitors. Importantly, CDK4/6 phosphorylates and inhibits TSC2, leading to mTORC1 activation and stimulating anabolism and cell growth, ensuring cells have sufficient mass to undergo mitosis [164]. In addition, CDK4 also phosphorylates FLCN, facilitating mTORC1 recruitment to lysosomes and its activation [162]. MYC, which acts upstream of CDK4, suppresses catabolic endolysosomal pathways in proliferating cells by directly binding to the promoters of lysosomal and autophagy genes, competing with TFEB [165,166]. Nevertheless, CDK4 appears to be required to keep lysosomal function intact in cancer cells [162]. This is in line with previous studies showing that autophagy occurs preferentially during the G1 and S phases of the cell cycle, when CDK4 is active [167]. Thus, CDK4 appears to promote mTOR activity and cell growth and to repress the autophagy-lysosomal pathway in proliferating cells.

Inversely, the CDK inhibitor p27^Kip1^ was shown to induce autophagy in starved cells [168,169,170,171,172]. In response to nutrient deprivation, p27 was shown to relocalize to the cytoplasm [168,169,170]. In cancer cells, cytoplasmic p27 may associate with CDK4/cyclin D1 complexes and promote CDK4 activity [173]. This is supported by the presence of p27 in immunoprecipitated CDK4 complexes in serum and glucose-starved cells [168]. However, in amino acid deprived cells, p27 promotes autophagy independent of CDKs by directly interacting with LAMTOR1 on lysosomes and interfering with Ragulator assembly, thus participating in mTORC1 inhibition, which, in turn, promotes lysosomal activity in a TFEB-dependent mechanism [169]. Surprisingly, in the context of glucose starvation, p27 favors autophagy by a different mechanism as cytoplasmic p27 facilitates autophagic vesicle trafficking by promoting microtubule acetylation via the stabilization of the microtubule acetyltransferase ATAT1 (alpha-tubulin N-acetyltransferase 1) [170].

As cells progress through S and G_2_ phases, TFEB drives the expression of lysosomal genes [21], but lysosome activity must be repressed in cells undergoing mitosis to protect genome integrity [32,174]. This inhibition of autophagy occurs even under nutrient starvation or pharmacological inhibition of mTORC1 [175]. Therefore, decoupling the nutrient-sensing mTORC1 pathway during cell cycle progression seems essential for mitotic division. Several studies have shown that mTORC1 fails to localize on lysosomes during mitosis [32,176]. Mechanistically, CDK1, which promotes the G_2_/M transition and mitotic progression, phosphorylates the Raptor subunit of mTORC1, preventing its interaction with Rags and subsequent mTORC1 activation on lysosomes [32]. CDK1 also targets and inactivates Vps34 [32,174], which participates in amino acids sensing upstream of mTORC1 [140,141,144]. Furthermore, CDK1 phosphorylates TFEB at the same sites targeted by mTORC1, leading to cytoplasmic retention of TFEB and inhibition of its transcriptional activity regardless of nutrient status [32]. While most studies have shown that the lysosomal-autophagy pathway is repressed during mitosis, others have challenged this idea [154,176,177,178]. However, these studies relied on observing autophagic vesicles upon treatment with pharmacological inhibitors of lysosomal function for extended durations (up to 24 h) that largely exceed the length of mitosis (60–90 min), making the conclusions drawn from such experiments difficult to interpret [176,177]. Further studies using live imaging techniques are needed to investigate the dynamics and activity of the lysosomal compartment throughout the cell cycle to clearly establish its role in mitosis, but fine-tuning lysosomal mass and activity appears essential for cell cycle progression (Figure 6), especially for the completion of cell division.

The final stage of cell division is cytokinesis, during which daughter cells physically separate in a process called abscission. Autophagy levels increase during anaphase and telophase, and it is thought that a burst of autophagic activity is required for the final step of cytokinesis [175,179,180]. During mitosis, cyclin B is degraded at the metaphase/anaphase transition, inactivating CDK1 [181]. This releases TFEB inhibition, promoting lysosome biogenesis. It is tempting to speculate that this step allows the activation of lysosome function in order to complete abscission. This idea is supported by the fact that inhibition of lysosomal v-ATPase impairs cytokinesis and results in multinucleated cells [179]. Moreover, RhoA, which plays an essential role in cytokinesis, must be turned off to complete abscission, and this is regulated by kinases such as citron kinase and PKCε [182]. It is not clear whether these kinases control the turnover of RhoA in cytokinesis, but it was found that RhoA is sequestered in autophagolysosomes in a p62-dependent manner for degradation, and this is required to allow abscission [179]. Interfering with autophagosome formation via ATG5 knockdown results in expansion of the active RhoA zone, leading to cytokinesis failure and multinucleation [179]. The ESCRT (endosomal sorting complex required for transport) complex that plays an essential role in cytokinesis is also involved in autophagy [183]. Indeed, the ESCRT subunit CHMP2A is required for phagophore closure and autophagosome formation [184]. However, it remains to be determined whether the role of the ESCRT complex in autophagy is important for the completion of cytokinesis.

The midbody, a microtubule-rich structure that forms in the intercellular bridge between two daughter cells during cytokinesis, is essential to recruit proteins controlling abscission [185]. After abscission, midbody remnants are either degraded by autophagy, released into the extracellular space, or maintained and accumulated in cells, where they appear to play a role in regulating cell fate in normal and cancer stem cells [186,187,188]. Although the exact mechanism that controls midbody removal via lysosomal pathways is still not fully elucidated, recent evidence points to an important role of the HIPK2 kinase, whose inhibition leads to midbody remnant accumulation and correlates with decreased levels of the autophagy receptors NBR1 and p62/SQSTM1 [189]. In another study, FYCO1, an LC3B partner, was found to be necessary for the formation of LC3B-containing membranes around midbody remnants and FYCO1 silencing leads to midbody accumulation [190]. In cells that accumulate midbody remnants, these are protected from lysosomal degradation by the formation of dynamic actin coats [191]. Interestingly, midbody accumulation has been observed in stem cells and cancer cells, and has been implicated in pluripotency and stemness, suggesting that lysosomes are implicated in cell commitment decisions in undifferentiated cells [186,188,191].

## 6. Role of Lysosomes in Stem Cell Metabolism and Fate

The roles of lysosomes in the regulation of the cell cycle have consequences for stem cells, affecting their potency, self-renewal capacity, and commitment decisions.

### 6.1. Role of the Lysosomal Machinery in Embryonic Stem Cells (ESCs)

Pluripotent embryonic stem cells (ESCs) originate from the inner cell mass of the epiblast and can differentiate in any cell type of the body. While pre-implantation ESCs are in a naïve state of pluripotency, post-implantation ESCs acquire a primed pluripotent state, with a propensity to differentiate towards specific lineages [192,193,194]. The transition between these states involves changes in their gene expression profiles, epigenetic marks, metabolism, and morphological features [192,193,194]. Surprisingly, the lysosomal machinery plays an important role in the conversion from naïve to primed pluripotency during development. For instance, proteins involved in the control of RagC/D activation on lysosomes, such as FLCN and TSC, are required to exit the naïve pluripotency state [66]. Active RagC/D is responsible for nuclear exclusion of TFE3, which precedes ESC priming [66,195]. Accordingly, the knockdown of Ragulator subunits (LAMTORs), which acts as a lysosomal scaffold for Rags, leads to nuclear accumulation of TFE3 and impairs ESC differentiation [66]. TFE3 drives the expression of ESRRB and WNT genes that maintain naïve pluripotency in mouse and human ESCs, respectively [195,196]. This effect is at least partially dependent on mTORC1 as rapamycin treatment rescues the differentiation block in TSC but not FLCN depleted cells [195]. Indeed, previous studies reported that mTOR is required for ESC proliferation and growth in early embryos by stabilizing the pluripotency transcription factors OCT4, NANOG, and SOX2, which repress the expression of developmentally regulated genes [197,198].

**Figure 7:**
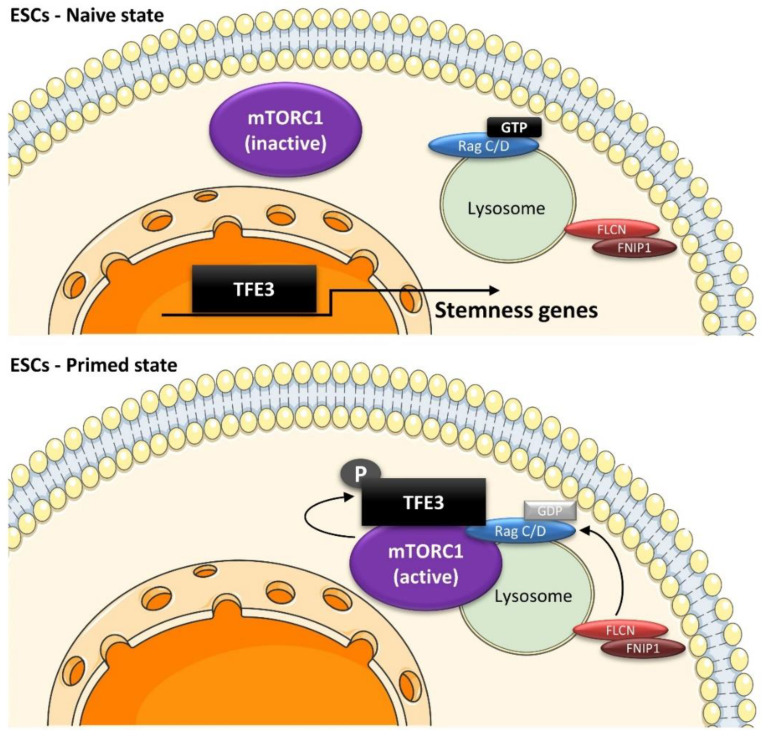
Role of the lysosomal machinery in ESC pluripotency. ESCs are maintained in a naïve state by nuclear TFE3, which induces expression of stemness-related genes, such as WNT in human ESCs or ESRBB in mouse ESCs. In response to differentiation signals, the FLCN–FNIP complex activates RagC/D, leading to TFE3 translocation to lysosomes and its phosphorylation by mTORC1. This causes cytoplasmic retention of TFE3 away from the nucleus and inhibition of its transcriptional activity, allowing ESCs to exit the naïve state of pluripotency.

During embryonic development, AMPK is required for endoderm differentiation via a TFEB-dependent mechanism. In AMPK^−/−^ embryos, hyperactivation of mTORC1 inhibits TFEB-driven lysosome biosynthesis. Normally, lysosomes help maintain WNT activity by sequestering GSK3β. In the absence of AMPK, free GSK3β inhibits β-catenin activity, preventing induction of WNT target genes and endoderm specification [199]. In a positive feedback loop, GSK3β directly phosphorylates TFEB (Figure 1), leading to its sequestration and inhibition on lysosomal membranes [29]. Interestingly, the reduction of cytoplasmic Ca^2+^ is required for mouse ESCs’ exit from naïve pluripotency [200], possibly via a Ca^2+^-dependent activation of TFEB [34]. Altogether, the lysosomal machinery plays a crucial role in the early stages of development and in controlling ESC fate and differentiation (Figure 7).

### 6.2. Role of the Lysosomal Machinery in Adult Stem Cells

Adult stem cells constitute rare populations of undifferentiated cells in most adult tissues. In contrast to ESCs, which are pluripotent, adult stem cells give rise to a limited number of mature cell types that build the tissue in which they reside. Most adult stem cells remain in a quiescent (G_0_) state characterized by low metabolic activity under normal conditions, but activating signals may trigger their exit from dormancy to proliferate and differentiate to regenerate the tissue. Cell cycle re-entry requires adjustments in nutrient uptake and metabolic pathways to meet increased bioenergetic needs. Conversely, these events must be reverted when these cells re-enter quiescence. Lysosomes play an important role in shaping the metabolic plasticity of adult stem cells in various tissues.

**Figure 8:**
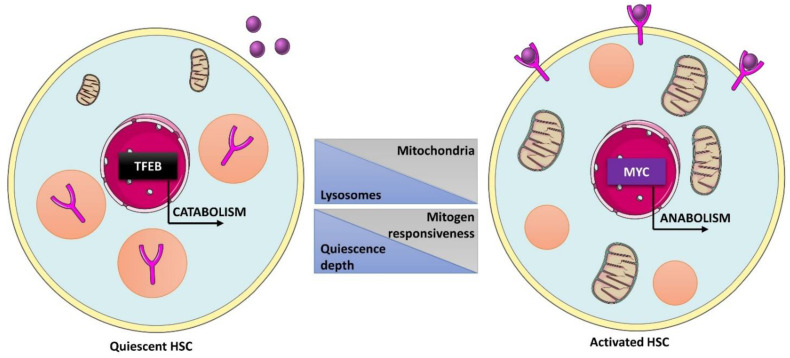
Role of lysosomes in human hematopoietic stem cells (HSCs) fate decisions. In quiescent HSCs, enhanced TFEB activity promotes catabolic pathways and lysosome-mediated degradation of membrane receptor involved in the response to mitogen signals, contributing to maintenance of a dormant state. Upon activation, MYC antagonizes TFEB activity, leading to expression of cell cycle, biosynthesis, and mitochondria-related genes, allowing cells to respond to mitogen stimuli by activation of anabolic pathways and subsequent cell cycle re-entry.

Quiescent hematopoietic stem cells (HSCs) are enriched in lysosomes and express high levels of lysosomal genes but exhibit low lysosomal activity, and these features are required to maintain quiescence and potency (Figure 8) [156,166]. Mechanistically, TFEB-mediated activation of lysosomal genes leads to degradation of TFR1 (membrane transferrin receptor 1), which uptakes ironbound transferrin, causing unresponsiveness of cells to mitogenic signals and sustaining a hypo-metabolic quiescent state [166]. Exit from dormancy is achieved when MYC counteracts TFEB activity, allowing cells to shut down their catabolism and induce anabolism by upregulating mitochondrial and pro-proliferative genes [166]. Importantly, TFEB was shown to drive the expression of myeloid-associated genes and to inhibit the expression of transcription factors associated with erythrocyte and megakaryocyte differentiation, such as GATA1 or RUNX1. Thus, TFEB overexpression results in a myeloid bias, whereas its silencing pushes cells towards erythroid differentiation. In fine, the balance between TFEB and MYC activity is a crucial determinant of human HSC metabolism and fate [166].

Interestingly, lysosomes are asymmetrically inherited by HSC progeny [201]. This is mediated by the NOTCH modulator NUMB, which partially colocalizes with lysosomes and is also asymmetrically co-inherited [201]. Cells with asymmetrically inherited lysosomes maintain a metabolically inactive state and exhibit higher overall heterogeneity in long-term differentiation without any bias for specific lineages [201]. In contrast, cells with lower lysosome content are predisposed towards the myeloid lineage [156]. During the transition from HSCs to myeloid progenitors, mTOR is targeted for proteasome-dependent degradation by the E3 ubiquitin ligase c-Cbl [202]. As a result, the translation of mTOR-dependent targets is reduced in multipotent progenitors compared to HSCs despite increased global translation [201,202]. Furthermore, quiescent HSCs are more reliant on oxidative phosphorylation than cycling HSCs, which require glycolysis [156]. In cycling-primed HSCs, pharmacological inhibition of either glycolysis, lysosomal activity, or mTORC1 signaling balances lineage production, indicating that lysosomal pathways coordinate the metabolic switch during HSC cell cycle re-entry and fate decisions [156].

Similarly, neural stem cells (NSCs) require TFEB-mediated activation of lysosomes to prime the conversion of quiescent NSCs into transit-amplifying cells and ultimately into functional neurons [203]. NSCs maintain quiescence by lysosomal-mediated degradation of environment-sensing receptors, thus relying on lysosomal activity to maintain a low metabolic state [204]. However, a reduction in the lysosomal activity in quiescent NSCs was also reported [203]. These differences may be associated with the age of the mice used in experiments as the expression of lysosomal and autophagy genes changes significantly with age in quiescent NSCs [205]. In old mice, systemic treatment with the mTOR inhibitor rapamycin activates TFEB and promotes priming of quiescent NSCs [203]. In contrast, in newborn mice, CSP-α (cysteine string protein-α) knockout causes hyperproliferation of radial glia-like NSCs due to mTOR activation and progressive exhaustion of NSCs in the hippocampus, and rapamycin treatment restores NSC quiescence [206]. Interestingly, CSP-α may be dynamically associated with the lysosome, depending on nutrient levels and mTORC1 activity status, suggesting that lysosomal proteins control the proliferation rate of NSCs [206].

TFEB-driven lysosome biosynthesis is also essential for lineage commitment in liver stem-like/progenitor cells that are responsible for liver regeneration [207]. These progenitors are normally bipotential, giving rise to hepatocytes or cholangiocytes, but they preferentially differentiate into hepatocytes after TFEB depletion [207].

An important role of lysosomes in the regulation of satellite cells, which are responsible for skeletal muscle regeneration, has been described in Pompe disease, which is characterized by skeletal muscle weakness and serious motor dysfunctions [208]. The disease is caused by glycogen accumulation due to deficiency of the lysosomal α-glucosidase (GAA), resulting in lysosome dysfunctions and myofiber death as satellite cells fail to repair disease-associated muscle damage [208]. Overexpression of TFEB and TFE3 in GAA-deficient muscle cells prevents glycogen accumulation and reduces lysosomal content by inducing exocytosis of autophagic vesicles, which seems promising to treat lysosomal storage disorders [209,210]. Lysosomes regulate satellite cell proliferation via an mTORC1-dependent mechanism [158]. First, the cMet-mTORC1 signaling axis is required to activate satellite cells upon injury, and Raptor knockout interferes with injury-induced exit from quiescence [158]. Second, SPAR (small regulatory polypeptide of amino acid response), a 90-aa peptide encoded by the long non-coding RNA LINC00961, interacts with v-ATPase subunits and prevents amino acid-mediated translocation of mTORC1 to lysosomes and activation [205]. SPAR depletion leads to mTORC1 hyperactivation and improves regeneration following injury [211]. Activated mTORC1 phosphorylates PASK (Per–Arnt–Sim domain kinase), which promotes WDR5-mediated epigenetic activation of the *Myogenin* promoter, resulting in the exit of satellite cells from self-renewal and initiation of myogenesis [212]. In addition, during late-stage myogenesis, mTORC1 acts via S6K1 to promote myoblast fusion [212]. In line with this, leucine and lysine supplementation, which activate the mTORC1/S6K1 pathway, promote skeletal muscle regeneration upon injury [213,214], possibly by facilitating amino acids incorporation in the damaged tissue [215,216]. This is consistent with the increased size of activated satellite cells compared to the quiescent population [158]. Finally, autophagy is essential for satellite cell activation by providing the nutrients necessary to meet bioenergetics needs during the transition from quiescent to active state [217], and autophagy impairment causes irreversible cell cycle arrest of satellite cells [218]. Since mTORC1 inhibits autophagy, it is unlikely that both pathways are activated at the same time in satellite cells following injury. A likely scenario is that autophagy is induced early on during satellite cell activation, providing the energy required for entering the cell cycle. Then, amino acids released from autolysosomes following autophagic degradation may activate mTORC1, leading to protein synthesis and muscle regeneration. In this scenario, lysosomes would coordinate the complex response of satellite cells, leading to skeletal muscle repair. Alternatively, mTORC1 and autophagy activation may occur simultaneously in distinct cellular compartments, as reported in the case of Ras-induced senescence [219].

The lysosomal machinery also controls intestinal stem cell (ISC) fate. ISCs reside in intestinal crypts and are responsible for the homeostatic renewal of the intestinal epithelium every 3 to 5 days and for intestinal epithelial repair after injury [220,221]. There are at least two types of ISCs: rapidly cycling crypt base columnar cells (CBCs, Lgr5^+^) located at the base of the crypt, and putative quiescent +4 ISCs that serve as a reserve population and can regenerate CBCs after damage [221]. Activation of the lysosome–autophagy pathway was shown to promote the regenerative capacities of CBCs in mice [222] and Drosophila [223] without significantly altering daily epithelium turnover [222]. The EphB3 receptor tyrosine kinase, which regulates intestinal cell positioning, is expressed as a gradient, being high in crypts and low in villi, controlled by lysosomes. Inhibition of lysosomal functions alters the EphB3 gradient and promotes ISC proliferation [224]. In Drosophila, loss of UVRAG, which regulates autophagy and endosome/lysosome trafficking and maturation, causes ISC hyperproliferation and dysplasia without affecting autophagy in these cells, suggesting that the endocytic function of UVRAG is important to control ISC behavior [225]. In line with this, the sorting nexin SH3PX1 restrains ISC proliferation through the endocytic network that regulates EGFR recycling to the plasma membrane [226]. Finally, the ARF1 GTPase involved in vesicle trafficking is essential for ISC homeostasis as ARF1 knockdown selectively kills ISCs through necrosis by inhibiting lipolysis without affecting differentiated cells [227]. This is due to the reliance of ICSs on lipid droplets as an energy source, whereas differentiated cells mainly use glucose and amino acids [227].

### 6.3. Role of the Lysosomal Machinery in Cancer Stem Cells

Cancer stem cells (CSCs) constitute small populations of undifferentiated tumor cells that share characteristics of somatic stem cells, such as self-renewal and quiescence, conferring them resistance to chemotherapy. A number of studies have shown that suppressing lysosomal functions helps targeting CSCs in various types of cancers, including breast [228,229], pancreas [230], lung [229], glioblastoma [231,232], and leukemia [233,234]. In pancreatic cancer, the addition of chloroquine to gemcitabine, a standard chemotherapeutic in pancreatic tumors, eliminates CSCs and causes tumor regression via inhibition of CXCL12/CXCR4 signaling, suppressing the ERK, STAT, and sonic hedgehog pathways in CSCs without affecting autophagy, suggesting that lysosomes directly modulate signal transduction in CSCs [230]. Furthermore, targeting of lysosomal iron translocation causes ROS accumulation and subsequent ferroptosis in CSCs of breast and lung cancer models [235]. Glioblastoma stem cells also undergo ferroptosis after treatment with lysosomal inhibitors in association with temozolomide [231]. Resistance to ferroptosis is a feature of metastasis-initiating cells (MICs), which have CSC properties [236]. Interestingly, MIC progenies are characterized by high TFEB expression and accumulation of lysosomes, which help them survive the metabolic stress they encounter during metastasis [237,238].

In fact, metabolic plasticity is one of the main hallmarks of CSCs. Persistent oxidative stress in tumor cells triggers a metabolic switch from glycolysis to the pentose phosphate pathway, which contributes to stemness-related features [239]. Lysosomal pathways limit ROS production by eliminating damaged organelles, especially mitochondria [152]. These studies suggest a direct relationship between lysosomes and CSC metabolism. Indeed, expression of the lysosomal/late endosomal marker LAMP1 correlates with low ROS levels in CSCs in colorectal cancer [229]. Furthermore, the inhibition of lysosomal functions by mefloquine reduces the levels of LAMP1/2 as well as the early and late endosome markers Rab5 and Rab7, respectively, leading to the elimination of CSCs via dysfunctional mitochondrial clearance in a colorectal cancer model [240]. Importantly, mefloquine did not affect expression of lysosomal and endosomal proteins in healthy cells, suggesting that lysosome targeting may be an effective strategy to selectively kill CSCs in colorectal cancer [240]. In glioblastoma, targeting lysosome synthesis by decreasing TFEB expression and activity with the HDAC inhibitor vorinostat in combination with melatonin sensitizes CSCs to chemotherapy-induced apoptosis [241].

CSC dormancy is thought to be responsible for treatment resistance and the eventual relapse of cancer patients. Several studies have shown that mTORC1 drives the activation of quiescent CSCs. For instance, the knockdown of TBK1 (TANK binding kinase 1), a negative regulator of mTORC1, decreases prostate cancer stem-like cell number and drug resistance, while rapamycin induces cell cycle arrest and enhances chemoresistance [242]. However, in human squamous cell carcinoma, mTORC1 hyperactivation contributes to the survival of dormant cells [243]. Therefore, the impact of mTORC1 signaling on CSCs seems to be context-dependent. Finally, many reports show that CSCs’ nutrient metabolism is different from that of normal cells, suggesting that the lysosomal nutrient-sensing machinery is a promising target for CSC eradication [244,245,246].

## 7. Conclusions and Perspectives

The lysosome has emerged as a master coordinator of signals regulating cell growth, proliferation, and differentiation. In addition to the crucial role of the lysosomal-autophagy pathway in cell metabolism, recent studies highlight the role of lysosomal membranes as a platform for a broad range of proteins, including metabolic intermediates and cell cycle regulators. Thus, lysosomes are gaining attention as a multifunctional signaling hub and as a druggable target with enormous therapeutic potential, allowing to not only control cell metabolism and proliferation but also fate and survival decisions. The promising role of lysosome manipulation is reflected by the fact that lysosomotropic agents are currently in clinical trials for many different indications, including cancer, neurodegenerative, and metabolic diseases.

## Data Availability

Not applicable.
